# Survival benefit of oral systemic monotherapy in previously treated metastatic colorectal cancer: a meta-analysis

**DOI:** 10.1093/oncolo/oyag175

**Published:** 2026-05-08

**Authors:** Per Pfeiffer, Chiara Cremolini, Michel Ducreux, Pia Osterlund, Sarah Ronnebaum, Morodoluwa Akin-Fajiye, Victoria Paly, Luis Hernandez, Elena Elez

**Affiliations:** Department of Oncology, Odense University Hospital, 5000 Odense, Denmark; Department of Translational Research and New Technology in Medicine and Surgery, University of Pisa, 56126 Pisa, Italy; Department of Medical Oncology, Paris-Saclay University, Gustave Roussy and INSERM-U1279, Collective Invasion, 94800 Villejuif, France; Department of Oncology, Tampere University Hospital Comprehensive Cancer Center and University of Tampere, 33100 Tampere, Finland; Department of Oncology, Tema Cancer/GI-Cancer, Karolinska University Hospital, Comprehensive Cancer Center, SE-171 76 Solna, Sweden; PPD Evidera Health Economics and Market Access, Thermo Fisher Scientific, Wilmington, NC 28401, United States; PPD Evidera Health Economics and Market Access, Thermo Fisher Scientific, Wilmington, NC 28401, United States; Global Pricing, Value & Access; Global Oncology Health Economics & US HEOR—Oncology, Takeda Pharmaceuticals America, Inc., Cambridge, MA 02139, United States; Global Pricing, Value & Access; Global Oncology Health Economics & US HEOR—Oncology, Takeda Pharmaceuticals America, Inc., Cambridge, MA 02139, United States; Medical Oncology, Vall d’Hebron Institute of Oncology, 08035 Barcelona, Spain

**Keywords:** meta-analysis, metastatic colorectal cancer, systemic monotherapies, survival benefit

## Abstract

**Background:**

Guideline-recommended nontargeted systemic therapies for previously treated metastatic colorectal cancer (mCRC) include regorafenib, trifluridine/tipiracil, and fruquintinib, but no consensus exists on the definition of clinically meaningful improvements for later-line mCRC treatments.

**Materials and methods:**

Trials were identified from systematic searches in MEDLINE, Embase, and the Cochrane Library. Meta-analyses were performed to characterize overall survival (OS) and progression-free survival (PFS) improvements with systemic therapy vs placebo in previously treated mCRC. Meta-analyses were conducted using fixed-effect and random-effects (RE) frequentist models of difference in medians, hazard ratios (HRs), and 12-month restricted mean survival time (RMST).

**Results:**

Six randomized, placebo-controlled, phase III trials of 3277 patients comparing oral systemic monotherapies with placebo were analyzed. Using the RE model, the meta-analyzed OS estimate for oral systemic monotherapy vs placebo was 1.86 months (95% confidence interval [CI], 1.30-2.42) for the difference in medians, 0.69 (95% CI, 0.64-0.76) for HRs, and 1.25 months (95% CI, 0.69-1.82) for the difference in 12-month RMST. For PFS, meta-analyzed median improvement was 0.97 months (95% CI, 0.28-1.66), HR was 0.38 (95% CI, 0.30-0.47), and 12-month RMST difference was 1.90 months (95% CI, 1.41-2.39). Sensitivity analyses, excluding the FRESCO-2 trial due to prior treatment differences, confirmed the primary meta-analysis results.

**Conclusion:**

When assessing the clinical benefit of later-line mCRC treatments, the broad clinical picture, including individualized treatment goals, should be evaluated. Considering multiple survival measures in the later-line mCRC context, an incremental survival improvement with oral systemic monotherapy vs no active therapy is clinically meaningful.

Implications for Practice In this article, three methods were used to analyze improvements in survival across six randomized clinical trials comparing oral treatments (regorafenib, trifluridine/tipiracil, or fruquintinib) with placebo for colorectal cancer (CRC) that has spread to other organs (metastatic CRC) after at least two previous treatments. The meta-analysis showed that the oral treatments provided approximately 1-2 months’ extension of overall survival and of progression-free survival, compared to placebo. The article highlights the importance of considering different measures of survival and the broad clinical context, including individualized treatment goals, when evaluating the clinical meaningfulness of trial results and when making treatment decisions.

## Introduction

Colorectal cancer (CRC) is the third most common cancer in the world and is the second leading cause of cancer-related mortality.^1^ Globally, CRC often presents at an advanced stage, with approximately 15%–30% of patients with CRC having metastatic disease at diagnosis.^2–7^ Moreover, 20%-50% of patients with localized CRC at diagnosis eventually develop metastases.^7^^,^^8^ The prognosis of patients with metastatic CRC (mCRC) remains poor with a 5‑year survival rate of approximately 14%.^9–11^

Although metastasectomy or ablation is attempted with curative intent in some patients with metastatic disease, relapse will occur in up to 55%-80% of cases, and the majority are not eligible for curative treatment due to clinical or disease characteristics.^7^ Thus, most patients with mCRC will receive systemic therapy given with palliative intent. Primary systemic treatment for most patients is chemotherapy, with or without antivascular endothelial growth factor (VEGF) biologics (i.e., bevacizumab) or, if *RAS* is wild type and left-sided primary tumor location, antiepidermal growth factor receptor (EGFR) biologics.^7^^,^^12^^,^^13^ Patients with actionable molecular alterations can receive immune checkpoint inhibitors in case of deficient mismatch repair or encorafenib with an anti-EGFR biologic if the *BRAF*-V600E mutation (*BRAF* mt) is present.^7^^,^^13^ Second-line treatment includes combinations of biologics and cytotoxic therapies.^7^^,^^12^^,^^13^

For most patients, disease relapse or progression will occur, regardless of first- and second-line treatment.^14^^,^^15^ For these patients, approved later-line systemic treatment options that are recommended by clinical guidelines include the oral systemic therapies regorafenib, trifluridine/tipiracil (TAS‑102) (with or without intravenous bevacizumab), and fruquintinib.^7^^,^^13^ The monotherapies regorafenib, TAS-102, and fruquintinib gained United States Food and Drug Administration and European Medicines Agency approval between 2012 and 2024.^16–21^ The regulatory approvals of these oral systemic treatments were based on outcomes from clinical trials of patients with refractory mCRC, where statistically significant improvements in overall survival (OS) were observed with active treatment compared with placebo.^22–27^

The absolute differences in median OS between active treatment and placebo observed in these trials ranged from 0.70 to 2.73 months.^22–27^ These incremental increases in median OS may not meet commonly cited targets for clinically meaningful improvement.^28–31^ However, other measures such as hazard ratios (HRs) and outcomes focusing on disease control such as progression-free survival (PFS) and quality of life (QoL) are also considered when assessing whether a treatment provides a clinically meaningful benefit in later-line metastatic oncology settings.^28^^,^^29^^,^^32^^,^^33^

To gain a comprehensive understanding of the survival benefit of the approved later-line treatments for mCRC, this systematic literature review (SLR) and meta-analysis aimed to characterize the OS and PFS benefits associated with systemic therapy relative to placebo (ie, best supportive care [BSC] alone) for patients with previously treated mCRC.

## Methods

An SLR and meta-analysis were performed in accordance with the Preferred Reporting Items for Systematic Reviews and Meta-Analyses (PRISMA) 2020 statement guidelines.^34^

### Data sources and search strategies

The SLR was initially conducted with a broad scope to summarize the evidence available for the treatment efficacy of regulatory-approved systemic therapies in adult patients with mCRC who had been previously treated with first- and second-line therapies, including fluoropyrimidine-, oxaliplatin-, and irinotecan-based chemotherapy, anti-VEGF therapy, and, if *RAS* wild type, an anti-EGFR therapy, depending on availability at the time and location of the study. Searches were performed on March 21, 2023, updated on October 4, 2023, and updated again on June 6, 2024, using MEDLINE, MEDLINE In‑Process, Embase, Cochrane Database of Systematic Reviews, and Cochrane Central Register of Controlled Trials (via OvidSP). In addition, gray literature was searched to identify any evidence from conference abstracts, clinical trial registries, Health Technology Assessment reports, and European public assessment reports. The comprehensive search strategy is included in Tables S1-S4. The searches were limited to publications written in English and conference abstracts published in or after 2020; there was no date limit for full-text publications. Searches were validated via manual review of the bibliographies of the most recent, relevant SLRs.

### Study eligibility and data extraction

Each reference was screened by two independent reviewers using pre-agreed Population, Intervention, Comparator, Outcome, and Study design criteria (Table 1), with disagreements resolved by a third independent reviewer. Relevant data were extracted using a data extraction template designed in Microsoft Excel^®^. Outcomes of interest included OS and PFS. Study information, including patient characteristics, treatment characteristics, and safety data, was also extracted and summarized descriptively.

**Table 1.** Inclusion and exclusion criteria for the meta-analysis.

	Inclusion	Exclusion
**Population**	Patients with mCRC who have been previously treated (received ≥2 prior lines of systemic chemotherapies) with or are not considered candidates for available therapies, including fluoropyrimidine-, oxaliplatin-, and irinotecan-based chemotherapy, an anti-VEGF therapy, and, if *RAS* wild type, an anti-EGFR therapy	Not mCRC
**Interventions**	Systemically administered pharmacologic agents with regulatory approval: Fruquintinib Regorafenib TAS-102 ± bevacizumab Rechallenge with treatments including but not limited to panitumumab, cetuximab, FOLFOX, FOLFIRI, CAPOX, bevacizumab, or other chemotherapy/targeted therapy treatments	No systemic therapy
**Comparators**	Placebo/BSC alone	No placebo/BSC-only arm
**Outcomes**	OS and PFS	Study does not report efficacy data
**Study design**	Phase III RCT	Case reports or series; in vitro, ex vivo, animal or pharmacokinetic studies; pooled analyses of RCTs; not phase III RCT; systematic reviews; dose finding studies; review or opinion with no original data

Abbreviations: BSC, best supportive care; EGFR, epidermal growth factor receptor; mCRC, metastatic colorectal cancer; OS, overall survival; PFS, progression-free survival; RCT, randomized controlled trial; TAS-102, trifluridine/tipiracil; VEGF, vascular endothelial growth factor.

### Risk-of-bias assessment

The risk-of-bias for each included study was assessed using version 2 of the Cochrane Risk-of-Bias tool for randomized studies, which categorizes studies as having “low risk-of-bias,” “some concerns,” or “high risk-of-bias” based on assessment of the randomization process, deviations from the intended population, missing outcome data, outcome measurement, and selection of reported result(s).^35^

### Meta-analyses

The meta-analyses included only phase III randomized controlled trials (RCTs) of later-line systemic therapies with a placebo (BSC alone) arm to ensure balance between treatment arms and sufficient sample size. Meta-analyses of incremental difference in median OS and median PFS were conducted in line with standard practice by calculating a synthesized mean of the difference in medians between the active treatment and placebo arms across studies. To ensure incremental changes in OS and PFS were captured over the follow-up of the included studies, meta-analyses of the HRs for OS and PFS were also conducted.

**Figure 1.**
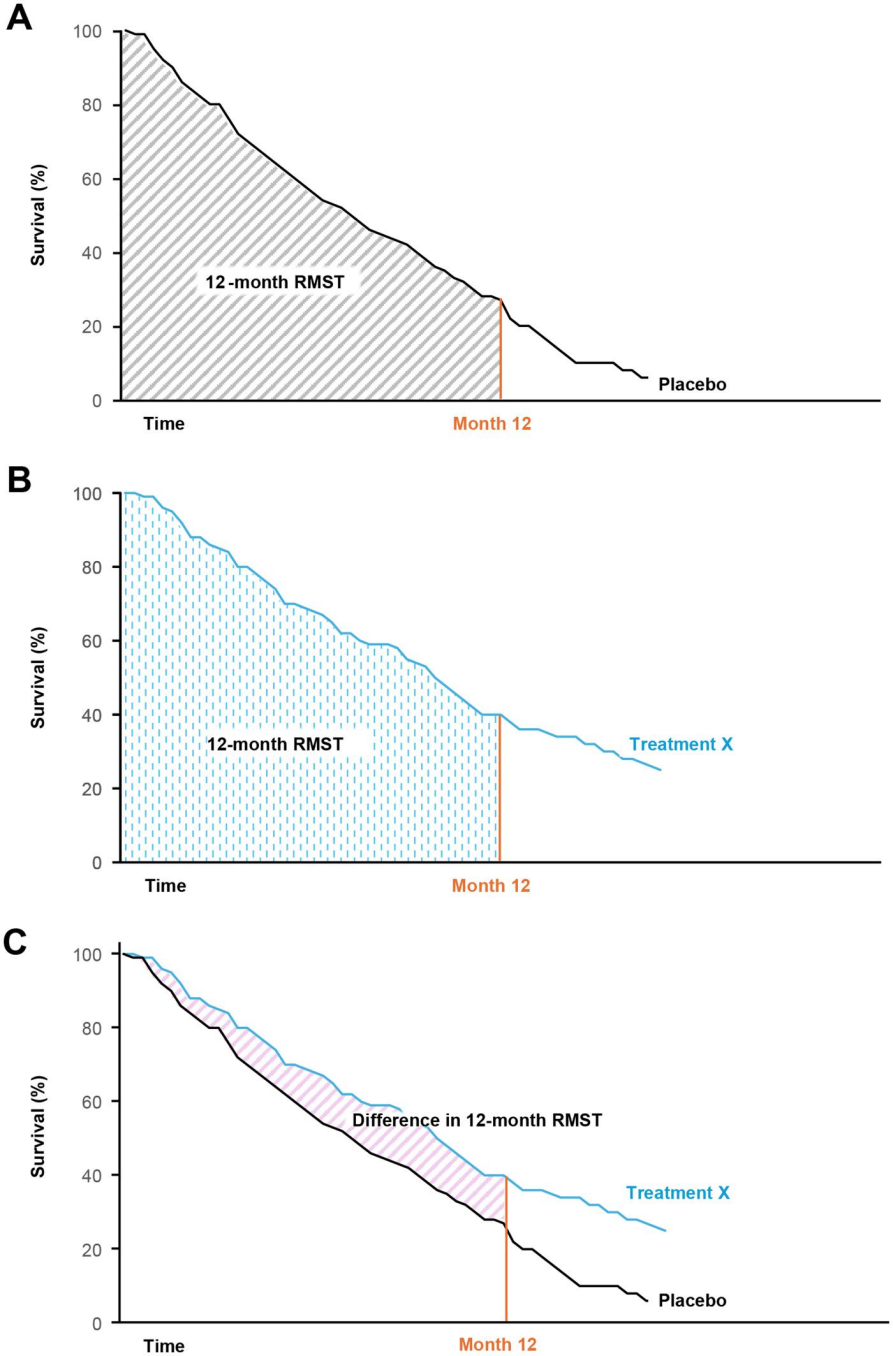
Schematic illustration of the difference in 12-month RMST calculation. Schematic figure (not real data) to illustrate calculation of difference in RMST between treatment arms at 12 months follow-up using the area under the K–M curve. The area under the K–M curve for the placebo arm up to the 12-month follow-up point (indicated by the shaded area) is subtracted from the equivalent area for the treatment arm to obtain the difference. The area(s) under the K–M curve(s) take into account the full 12‑month follow-up period, accounting for any changes in proportional hazards over time. K–M, Kaplan–Meier; RMST, restricted mean survival time.

Similar analyses were run for the difference in restricted mean survival time (RMST) between treatment arms at 12 months. RMST is another way to examine survival time, calculated as the area under the Kaplan–Meier curve between two time points (at baseline/time 0 and a landmark time point, in this case 12 months; Figure 1). The 12-month landmark time point was selected for the RMST meta-analyses based on the maturity of the OS and PFS Kaplan–Meier data across all included studies.

RMST can provide a useful alternative for assessing between-group differences in survival, for example, when the proportional hazards assumption is not met. The proportional hazards assumption states that the relative event rate between groups is constant throughout the follow-up period and is used when calculating HRs.^36^ However, the proportional hazards assumption does not hold true in all cases. When the proportional hazards assumption is violated, it can result in potential bias when interpreting HRs and differences in medians.^37^ Between-group differences in RMST can, therefore, provide a more clinically meaningful and interpretable measure of incremental survival benefit over the landmark time period in situations where the proportional hazard assumption does not hold.^38–40^

Fixed-effect (FE) and random-effects (RE) frequentist meta-analyses were conducted for OS and PFS, with each analysis estimating the mean effect and its standard error and 95% confidence interval (CI), in line with standard practice. Statistical heterogeneity was evaluated by assessing the test of homogeneity and consideration of the size of *I*^2^ and *τ*^2^. An $I^{2}$ value of 0% indicates no observed heterogeneity, and larger values show increasing heterogeneity.^41^ Typically, $I^{2}$ values of 25%, 50%, and 75% are considered low, moderate, and high heterogeneity, respectively. In RE models, *τ*^2^ represents the variance between studies, and when *τ*^2^ = 0, the RE model converges with the FE model. All meta-analyses were conducted with a restricted maximum-likelihood approach, using the *metafor* package (version 4.4)^42^ for the R software environment (version 4.4.1).

Where differences between study populations were identified, sensitivity analyses were performed in which the outlier study was excluded from each meta-analysis (difference in medians, HRs, and 12-month RMST).

## Results

### Studies included in the meta-analyses

**Figure 2.**
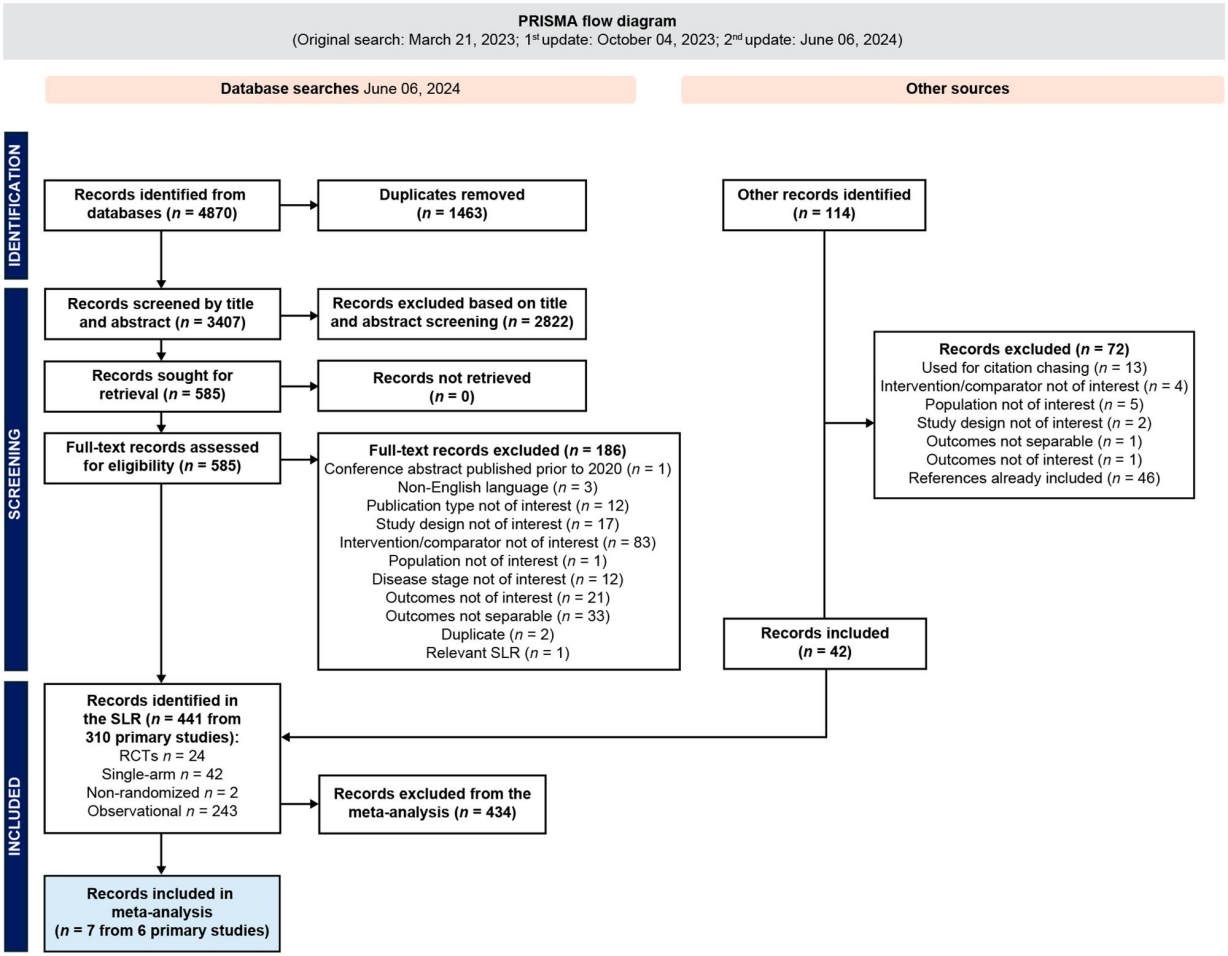
PRISMA diagram of study identification for the meta-analyses. Ten phase III RCTs and one phase II/III RCT were identified in the systematic literature search. Of these, six phase III RCTs with a placebo arm were identified for inclusion in the meta-analyses. RCT, randomized controlled trial; SLR, systematic literature review.

The systematic review identified a total of 4870 records in the database search, of which 1463 were removed as duplicates. In total, 3407 records were screened at the title and abstract level, identifying 585 records for full-text review for eligibility. Seven records representing six phase III placebo-controlled RCTs for systemic therapies with regulatory approval met the inclusion criteria for the meta-analyses (Table 1).^22–27^^,^^43^ One additional phase III RCT, SUNLIGHT, was also identified in the systematic review. SUNLIGHT compared TAS‑102 in combination with intravenous bevacizumab with TAS-102 monotherapy,^44^ but did not include a placebo arm and could not be included in the meta-analyses. The PRISMA diagram is presented in Figure 2.

All of the phase III placebo-controlled RCTs included in the meta-analyses evaluated oral systemic monotherapies. Two RCTs (CONCUR^24^ and CORRECT^23^) investigated regorafenib vs placebo, two RCTs (RECOURSE^25^^,^^43^ and TERRA^27^) investigated TAS-102 vs placebo, and two RCTs (FRESCO^26^ and FRESCO-2^22^) investigated fruquintinib vs placebo.

The RCTs included in this analysis were at a low risk-for-bias (Table S5).^35^

### Population and study characteristics

For the RCTs included in the meta-analyses, start dates for patient recruitment ranged from 2010 (CORRECT) to 2020 (FRESCO-2). Three studies were conducted in Asia only (CONCUR, FRESCO, and TERRA), and three were global trials (CORRECT, FRESCO-2, and RECOURSE).^22–27^ There were a total of 3277 participants across the trials. Median age ranged from approximately 55-64 years and the proportion of males ranged from approximately 49%-70%.^22–27^^,^^43^ Reporting and/or prevalence of disease characteristics, such as liver metastases, and molecular characteristics, such as the presence of *BRAF* mt and *KRAS/RAS* mutations, varied between trials. Study and population characteristics are presented in Table 2.

**Table 2.** Study characteristics, demographics, and disease characteristics.

Trial, start of enrollment	Region	Trial arm	*n*	Median age, years (range)	Male, %	Asian race, %	Asian geographic location, %	ECOG 0/1, %	Number of metastatic sites, %	*KRAS/RAS* mutation, *n* (%)	*BRAF* mt, *n/N* (%)	Liver metastasis, *n* (%)	Prior lines of treatment, %
**CONCUR, 2012^24^**	Asia	Regorafenib	136	58 (IQR, 50-66)	63	100	100	26/74	1: 21 ≥2: 79	Mutant: 46 (34)	Yes: 0 (0) No: 28 (21) Unknown: 108 (79)	NR	For metastatic disease: 1-2: 35^a^^,^^b^ 3: 24^a^ ≥4: 38^a^
		Placebo	68	56 (IQR, 49-62)	49	100	100	22/78	1: 22 ≥2: 78	Mutant: 18 (26)	Yes: 1 (1) No: 14 (21) Unknown: 53 (78)	NR	For metastatic disease: 1-2: 35^a^ 3: 25^a^ ≥4: 40^a^
**CORRECT, 2010^23^**	Global	Regorafenib	505	61 (IQR, 54-67)	62	15	14	52/48	NR	Mutant: 273 (54)	Yes: 14 (4) No: 322 (96)	NR	For metastatic disease: 1-2: 27^a^^,^^b^ 3: 25^a^ ≥4: 49^a^
		Placebo	255	61 (IQR, 54-68)	60	14	14	57/43	NR	Mutant: 157 (62)	Yes: 3 (2) No: 163 (98)	NR	For metastatic disease: 1-2: 25^a^^,^^b^ 3: 28^a^ ≥4: 47^a^
**FRESCO, 2014^26^**	Asia	Fruquintinib	278	55 (23-75)	57	100	100	28/72	1: 5 ≥2: 95	157 (57)	NR	185 (67)	For metastatic disease: ≤3: 79^a^ ≥4: 21^a^
		Placebo	138	57 (24-74)	70	100	100	27/73	1: 3 ≥2: 97	74 (54)	NR	102 (74)	For metastatic disease: ≤3: 78^a^ ≥4: 22^a^
**FRESCO-2, 2020^22^**	Global	Fruquintinib	461	64 (IQR: 56-70)	53	9	9 (Japan) 11 (Japan and Australia)	43/57	1: 13 ≥2: 87	291 (63)	Wild type: 401 (87) V600 E mutation: 7 (2) Other: 53 (11)	339 (74)	For metastatic disease: ≤3: 27^a^ ≥4: 73^a^
		Placebo	230	64 (IQR: 56-69)	61	8	7 (Japan) 10 (Japan and Australia)	44/56	1: 18 ≥2: 82	145 (63)	Wild type: 198 (86) V600 E mutation: 10 (4) Other: 22 (10)	156 (68)	For metastatic disease: ≤3: 28^a^ ≥4: 72^a^
**RECOURSE, 2012^25^^,^^43^**	Global	TAS-102	534	63 (27-82)	61	34	33	56/44	1-2: 61 ≥3: 39	Mutant: 272 (51)	NR	NR	Treatment intent not specified: 2: 18 3: 22 ≥4: 60
		Placebo	266	63 (27-82)	62	35	33	55/45	1-2: 58 ≥3: 42	Mutant: 135 (51)	NR	NR	Treatment intent not specified: 2: 17 3: 20 ≥4: 63
**TERRA, 2013^27^**	Asia	TAS-102	271	58 (26-81)	63	100	100	24/76	1-2: 61 ≥3: 39	Mutant: 99 (37)	NR	NR	Treatment intent not specified: 2: 23 3: 27 ≥4: 50
		Placebo	135	56 (24-80)	62	100	100	22/78	1-2: 61 ≥3: 39	Mutant: 50 (37)	NR	NR	Treatment intent not specified: 2: 19 3: 27 ≥4: 55

Abbreviations: *BRAF* mt, *BRAF* V600E mutation; ECOG, Eastern Cooperative Oncology Group; IQR, interquartile range; NR, not reported; TAS-102, trifluridine/tipiracil.

a Prior lines of therapy in metastatic disease.

b In CONCUR, four patients (3%) in the regorafenib group had not previously received any treatment for metastatic disease;^24^ in CORRECT, five patients (2%) in the placebo group and 16 patients (3%) in the regorafenib group had received only one previous line of therapy for metastatic disease.^23^

All studies included in the meta-analyses required patients to have received either ≥2 lines of standard chemotherapies for advanced disease (fluoropyrimidine-, oxaliplatin-, and irinotecan-based chemotherapy) or to have received all current locally approved standard therapies. Patients may have also received anti-VEGF therapy and, if *RAS* wild type, an anti-EGFR therapy. Data on prior therapies received by patients in the studies included in the meta-analyses are shown in Table 2 and Table S6.

CORRECT (2010) and CONCUR (2012) included approximately 25% and 35% of patients who had received 1-2 prior therapy lines, respectively.^23^^,^^24^ Patients enrolled in RECOURSE, TERRA, and FRESCO-2 had received more prior therapies; ≥4 prior therapies had been received by ∼51% of patients in TERRA, ∼61% of patients in RECOURSE, and ∼73% of patients in FRESCO-2.^22^^,^^25^^,^^27^ Almost all patients (∼96%-100%) in the global trials RECOURSE, FRESCO-2, and CORRECT had been previously treated with an anti-VEGF therapy, compared with ∼30%-39% in the Asia-only trials CONCUR, FRESCO, and TERRA.^22–27^^,^^43^ Prior treatment with anti-EGFR therapy varied between the studies from ∼14% in FRESCO to ∼53% in RECOURSE.^22–27^^,^^43^ Approximately 20% of patients in RECOURSE had received prior regorafenib.^25^ FRESCO-2 was the only study requiring prior regorafenib and/or TAS-102; ∼53% of patients were previously treated with TAS-102, ∼9% with regorafenib, and ∼40% with both TAS-102 and regorafenib.^22^

### Overall survival

**Figure 3.**
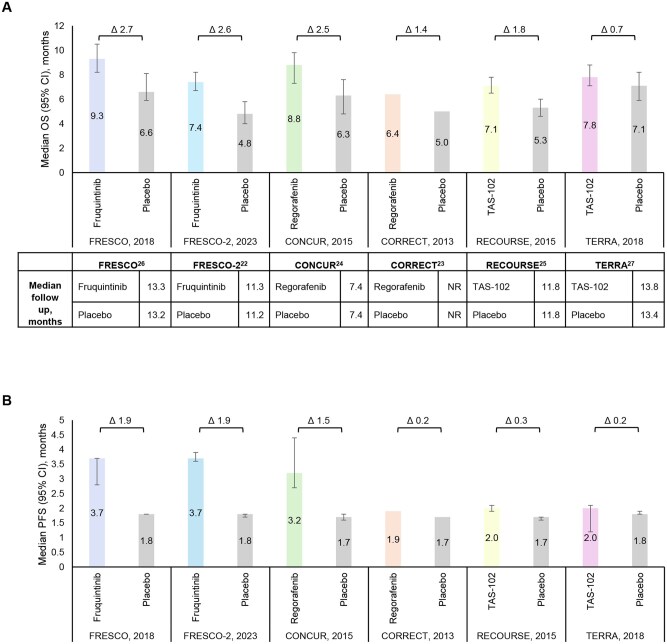
Absolute median outcomes in months (95% CI) with active treatment vs placebo for (A) OS and (B) PFS. 95% CIs were not reported in CORRECT. Δ, difference in medians between active treatment vs placebo. CI, confidence interval; OS, overall survival; PFS, progression-free survival; TAS-102, trifluridine/tipiracil.

The absolute median OS values for the placebo arms across all studies ranged from 4.8 to 7.1 months (Figure 3A). Statistically significant improvements in OS were reported with oral systemic monotherapies vs placebo across all studies, according to Kaplan–Meier analysis.^22–27^ The largest difference in median OS between oral systemic monotherapy and placebo was reported in FRESCO (fruquintinib; 2.7 months)^26^ followed by FRESCO-2 (fruquintinib; 2.6 months),^22^ CONCUR (regorafenib; 2.5 months),^24^ RECOURSE (TAS-102; 1.8 months),^25^^,^^43^ CORRECT (regorafenib; 1.4 months)^23^ and TERRA (TAS-102; 0.7 months).^27^

**Figure 4.**
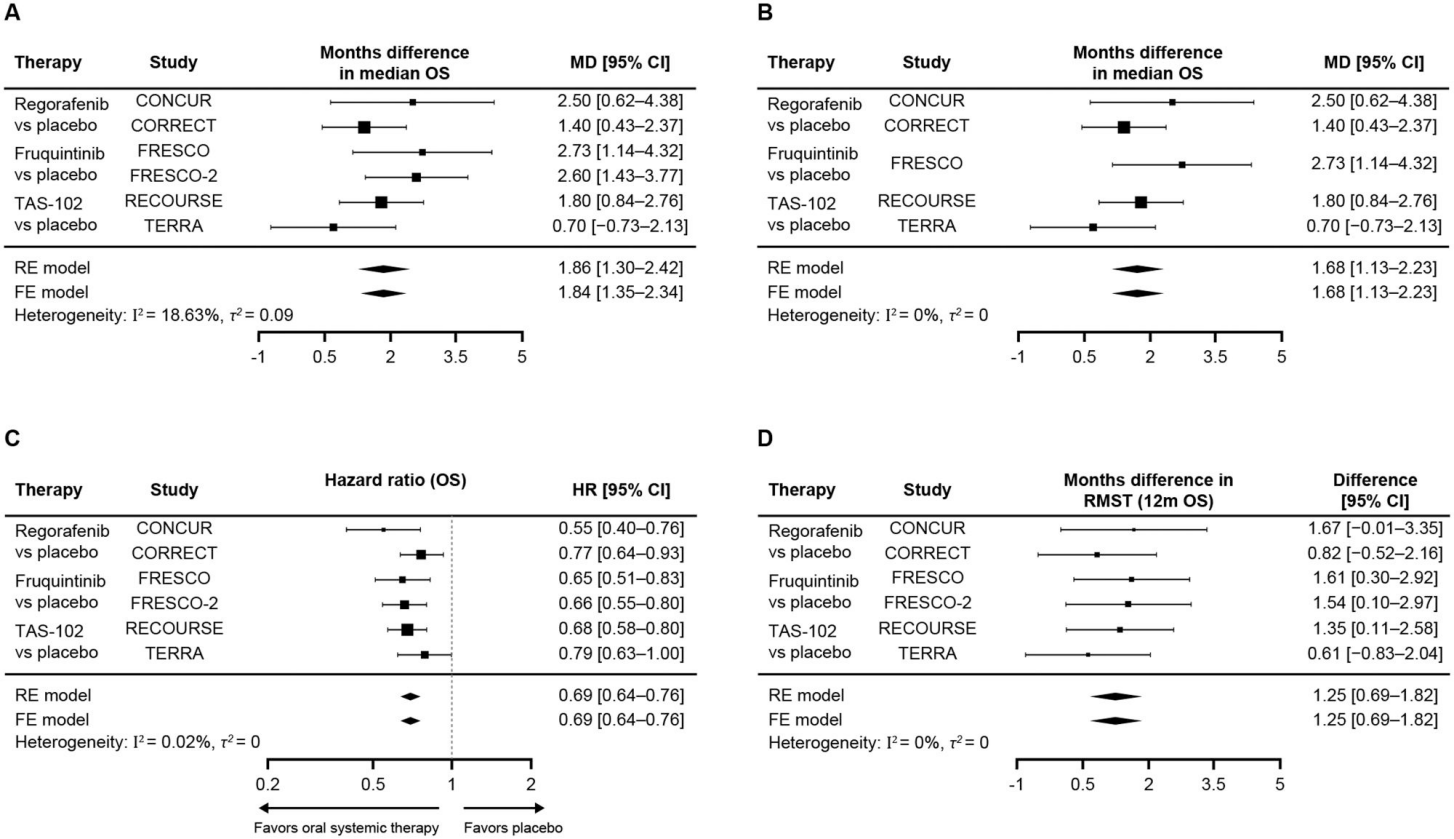
Meta-analyses of difference in (A) median OS, (B) median OS, excluding FRESCO-2 (sensitivity analysis), (C) HR for OS, and (D) 12-month RMST for OS. CI, confidence interval; FE, fixed effect; HR, hazard ratio; MD, mean difference; OS, overall survival; RE, random effects; RMST, restricted mean survival time; TAS-102, trifluridine/tipiracil.

Meta-analysis of incremental survival benefit in terms of median OS in months showed statistically significant OS benefit for oral systemic monotherapies vs placebo in both the RE model (1.9 months [95% CI, 1.3-2.4]) and the FE model (1.8 months [95% CI, 1.4-2.3]) (Figure 4A). There was a low level of heterogeneity among the studies analyzed ($I^{2}$ = 18.63%). The assumption of homogeneity in the underlying effect sizes was confirmed by the similarity in the RE and FE model point estimates of 1.9 and 1.8, respectively (Figure 4A). As FRESCO-2 was the only study requiring prior treatment with, or intolerance to, TAS‑102 or regorafenib, and patients had received more prior treatments than those enrolled in the other included studies,^22^ a sensitivity analysis was performed excluding FRESCO-2. When FRESCO-2 was excluded, the improvement in median OS for oral systemic monotherapies vs placebo across all the remaining studies was 1.7 months (95% CI, 1.1-2.2) using the RE model and 1.7 months (95% CI, 1.1-2.2) using the FE model, with heterogeneity decreased to 0% (Figure 4B).

Statistically significant improvement in OS with oral systemic monotherapies vs placebo was also seen in the meta-analysis of HRs. The risk of death was reduced by 31% (HR 0.69 [95% CI, 0.64-0.76]) in both the RE and FE models, with an $I^{2}$ value of 0.02%, showing homogeneity in the results (Figure 4C). Consistent results were seen in the sensitivity analysis excluding FRESCO-2 from the meta-analysis of HRs (Figure S1A).

In the meta-analysis of 12-month RMST for OS, oral systemic monotherapies were also associated with an incremental survival increase of 1.3 months (95% CI, 0.7-1.8) relative to placebo in both the FE and RE models, with an $I^{2}$ value of 0%, confirming homogeneity across studies (Figure 4D). Consistent results were seen in sensitivity analysis excluding FRESCO-2 from the meta-analysis of 12-month RMSTs (Figure S1B).

### Progression-free survival

Absolute median PFS values for the placebo arms were consistent across all studies, ranging from 1.7 to 1.8 months. Statistically significant improvements in PFS were also reported with oral systemic monotherapies vs placebo across studies in Kaplan–Meier analyses.^22–27^ The highest difference in median PFS was recorded in FRESCO and FRESCO‑2 (fruquintinib; both 1.9 months),^22^^,^^26^ followed by CONCUR (regorafenib; 1.5 months),^24^ RECOURSE (TAS-102; 0.3 months),^25^^,^^43^ CORRECT (regorafenib; 0.2 months),^23^ and TERRA (TAS-102; 0.2 months)^27^ (Figure 3B).

**Figure 5.**
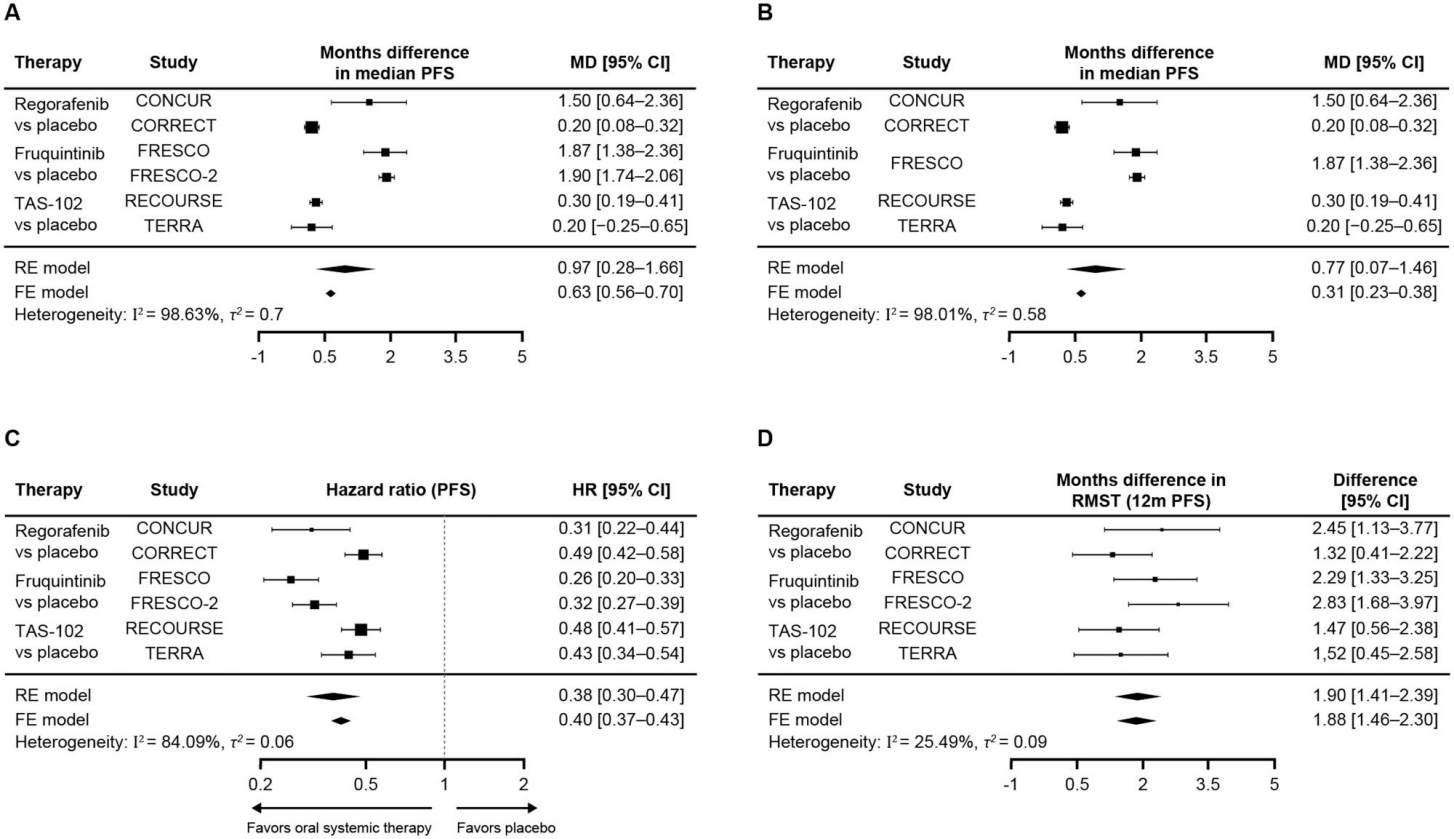
Meta-analyses of difference in (A) median PFS, (B) median PFS excluding FRESCO-2 (sensitivity analysis), (C) HR for PFS, and (D) 12-month RMST for PFS. CI, confidence interval; FE, fixed effects; HR, hazard ratio; MD, mean difference; PFS, progression-free survival; RE, random effects; RMST, restricted mean survival time; TAS-102, trifluridine/tipiracil.

Meta-analysis of incremental PFS benefit in terms of median PFS in months is shown in Figure 5A. The incremental benefit with oral systemic monotherapies vs placebo was 1.0 months (95% CI, 0.3-1.7) in the RE model, compared to 0.6 months (95% CI, 0.6-0.7) in the FE model. There was a high level of heterogeneity among the studies analyzed ($I^{2}$ = 98.6%; Figure 5A). A statistically significant incremental survival improvement was seen in the sensitivity analysis excluding FRESCO-2 from the meta-analysis (0.8 months [95% CI, 0.1-1.5] in the RE model and 0.3 months [95% CI, 0.2-0.4] in the FE model), but heterogeneity remained high ($I^{2}$ = 98.0%; Figure 5B).

In the meta-analysis of HRs for PFS, the estimates for risk of progression or death with oral systemic monotherapies vs placebo were similar in the RE and FE models: 0.4 (95% CI, 0.3-0.5) in the RE model and 0.4 (95% CI, 0.37-0.43) in the FE model (Figure 5C). The HR results across studies were more homogenous than the difference in median PFS, but heterogeneity remained high ($I^{2}$ = 84.1%).

In the meta-analysis of 12-month RMST for PFS, the incremental difference in 12-month RMST for PFS was 1.9 months (95% CI, 1.4-2.4) in the RE model and 1.9 months (95% CI, 1.5-2.3) in the FE model in favor of oral systemic monotherapies vs placebo, with lower heterogeneity ($I^{2}$ = 25.5%; Figure 5D).

As with OS, results of sensitivity analyses excluding FRESCO-2 from the meta-analyses of HRs and 12-month RMSTs for PFS were consistent with the main meta-analyses (Figure S1C and D).

### Safety

Grade 3-4 adverse events (AEs) reported in the included trials are summarized in Figure S2, with the most frequently reported AEs being hematologic toxicities for TAS-102, hand-foot syndrome and fatigue for regorafenib, and hypertension for fruquintinib.^22–27^

## Discussion

The prognosis of patients with mCRC worsens as the disease progresses, and as many patients receive three or more lines of therapy, the clinical benefit of approved treatments should be assessed in the context of this poor prognostic, later-line disease setting.^45^ This systematic review and meta-analysis of phase III RCTs conducted in patients with previously treated mCRC showed incremental OS and PFS benefits for oral systemic monotherapy relative to placebo (BSC alone) across estimates of median survival, HRs, and 12-month RMST. Sensitivity analyses excluding the FRESCO-2 trial confirmed the results of the primary meta-analyses of statistically significant incremental OS and PFS benefits with oral systemic monotherapies vs placebo in the later-line mCRC setting.

Our findings highlight the importance of considering multiple measures of survival when assessing whether trial results are clinically meaningful, as well as the full clinical context. Published reviews have highlighted the lack of a consensus definition for clinically meaningful benefit in CRC, noting that recommended meaningfulness thresholds from American Society of Clinical Oncology (ASCO) and European Society for Medical Oncology (ESMO) are not consistently met in RCTs.^30^^,^^46^^,^^47^ The difference in median OS of 1.9 months for oral systemic monotherapies vs placebo in this meta-analysis does not meet the benchmarks of ≥2 months, ≥3 months, and 3-5 months set by Colorectal Cancer Canada (CCC), ESMO-Magnitude of Clinical Benefit Scale (MCBS) (Grade 4 if median OS with standard treatment is <12 months), and ASCO-CRC, respectively,^28–31^ with three of the six individual trials reporting incremental OS improvements of <2 months (Figures 3 and 4). The poor prognosis of patients with mCRC treated in the later-line setting should be considered, along with individualized treatment goals. In the placebo arms of the included RCTs, OS ranged from 4.8 to 7.1 months; in this context, an improvement below 2 months could be considered clinically meaningful for many patients. Moreover, median values are point estimates and may not reflect between-treatment differences over the full follow-up period. As mCRC is heterogeneous, with not all patients experiencing a clinical benefit from treatment, especially in the later-line refractory mCRC setting,^7^ relative measures of survival benefit that take into account the full follow-up period (i.e., HRs) may, therefore, be more relevant than benchmarks based on absolute measures like median survival.

As well as benchmarks for median survival improvements, ESMO-MCBS, ASCO, and CCC provide thresholds for HRs,^28^^,^^29^^,^^31^ with the ESMO-MCBS grading system recommending consideration of both HRs and median survival.^31^ The recommended OS thresholds are ≤0.65 (ESMO-MCBS Grades 2-4, depending on median OS gain),^31^ ≤0.67 (ASCO-CRC),^29^ and ≤0.75 (CCC).^28^ Following a statistical analysis, ESMO-MCBS recommends that the lower limit of the 95% CI is considered when assessing the clinical meaningfulness of HRs against this threshold.^48^ In this meta-analysis, the HR for OS was 0.69 (95% CI, 0.64-0.76) in both the RE and FE models, with the lower CI limits indicating clinically meaningful survival improvements for oral systemic monotherapies relative to placebo against these thresholds (Figure 4).

In terms of median PFS, ESMO-MCBS and ASCO-CRC also make recommendations for thresholds for meaningful improvements: ≥1.5 months (for Grade 3; when PFS is the primary endpoint)^31^ and 3-5 months, respectively.^29^ For the individual RCTs, the differences in median PFS between active treatment and placebo ranged from 0.2 to 1.9 months across the studies. In the meta-analysis, there was a high degree of heterogeneity and variation between the RE and FE estimates (point estimates of 1.0 months and 0.6 months, respectively [Figure 5]). ESMO-MCBS also provides an HR threshold for PFS of ≤0.65 (if PFS is the primary endpoint: Grade 3 if median gain ≥1.5 months; Grade 2 if median gain <1.5 months).^31^ In the meta-analysis, HRs for PFS were 0.38 (95% CI, 0.30-0.47) in the RE analysis and 0.40 (95% CI, 0.37-0.43) in the FE analysis, meeting this ESMO-MCBS threshold. There was also high heterogeneity in meta-analyses of the difference in median PFS ($I^{2}$ = 99%) and HRs for PFS ($I^{2}$ = 84%) for oral systemic monotherapies vs placebo. Despite this heterogeneity in the meta-analyses, placebo PFS outcomes were highly consistent across the individual RCTs (range 1.7-1.8 months). This consistency may support ongoing efforts to develop external or synthetic control arms in mCRC, such as those undertaken by the ARCAD consortium;^49^ however, such approaches typically require larger datasets and were beyond the scope of this analysis.

In addition to median survival and HRs, mean survival times such as RMST can be a useful alternative metric for characterizing survival benefit, particularly in the context of mature survival data and in cases where the proportional hazards assumption may be violated. Because Kaplan–Meier curves for both OS and PFS were nearly complete across all of the studies included in the meta-analyses, RMST at 12 months could be calculated for all studies. The meta-analysis for the difference in 12‑month RMST for OS showed that patients receiving oral systemic monotherapy survived on average (mean) 1.3 months longer within 12 months of follow-up (95% CI, 0.7-1.8) in both the FE and RE analyses for oral systemic monotherapy relative to placebo (Figure 4). The meta-analyzed difference in 12-month RMST for PFS showed that patients did not experience disease progression or die for an average of 1.9 months longer with oral systemic monotherapy relative to placebo (Figure 5). These 12-month RMST findings were consistent with the results of the meta-analyses of differences in medians and HRs. In addition, heterogeneity was reduced in the difference in 12-month RMST meta-analyses, particularly for PFS, where $I^{2}$ was 26%, compared with 99% and 84% for the difference in medians and HRs, respectively. These results support the use of RMST as an additional measure of survival benefit that can be considered alongside differences in medians and HRs when assessing the clinical meaningfulness of survival benefits in mCRC clinical trials. However, it is important to note that, unlike medians and HRs, there are no proposed thresholds for clinical meaningfulness of RMST. Any such benchmark would need to be conditioned on the time horizon used to calculate RMST and the completeness of the survival curves.

We acknowledge the following limitations of our study. First, this meta-analysis was not designed to indirectly compare the efficacy of the individual systemic therapies, which have different mechanisms of action and distinct safety profiles. While population-adjusted methods and indirect comparisons have been used to evaluate relative efficacy between individual treatments in published network meta-analyses,^50–52^ this meta-analysis was designed to characterize the survival benefit of systemic therapies vs placebo across studies. Therefore, no population matching was conducted, and any potential imbalances between treatment arms of individual trials were not accounted for. There was heterogeneity in the population characteristics between studies, particularly in the prior treatments received and geographic location/race (Asian vs non-Asian studies); reporting of molecular and disease characteristics such as liver metastases also varied between studies (Table 2 and Table S6). Differences in the number of prior treatments received likely reflect the trial geography and the broad period over which the included trials were initiated; start dates of recruitment ranged from 2010 to 2020, a time period in which regorafenib and TAS-102 were introduced to the later-line mCRC treatment paradigm. Prior regorafenib and/or TAS-102 treatment was an inclusion criterion in FRESCO-2,^22^ accounting for the higher number of prior treatments received by patients in the study population compared with the trials initiated at earlier time points. Additionally, a lower proportion of prior anti-VEGF use was observed in the Asia-only trials compared to the global trials. Moreover, several potential effect modifiers were identified in interaction tests of subgroup HR results in the individual trials. For OS, these included prior targeted biological treatment (CONCUR)^24^ and age (TERRA).^27^ For PFS, these included *KRAS* mutation (FRESCO),^26^ prior targeted biological treatment (CONCUR),^24^ sex (CONCUR),^24^ and age (CORRECT).^23^ Unfortunately, more granular subgroup analyses by specific line of therapy or other characteristics were not feasible due to differences in how these were defined and reported across studies.

The meta-analyses confirmed a statistically significant OS and PFS benefit for oral systemic monotherapies vs placebo across these heterogeneous study populations. For the difference in median OS vs placebo, there was heterogeneity between the regorafenib trials (CONCUR: 2.5 months, CORRECT: 1.4 months)^23^^,^^24^ and the TAS‑102 trials (RECOURSE: 1.8 months, TERRA: 0.7 months)^25^^,^^27^ but the median OS benefit observed with fruquintinib was consistent between both trials (FRESCO: 2.7 months; FRESCO-2: 2.6 months),^22^^,^^26^ despite the greater number of prior therapies received by the population in FRESCO-2 compared with FRESCO (Figure 4).

The inclusion criteria for the meta-analysis were limited to only phase III clinical trials that compared systemic monotherapies to placebo, leading to the exclusion of SUNLIGHT, a phase III RCT assessing TAS-102 in combination with bevacizumab compared with TAS-102 alone.^44^ The absolute difference in median OS reported in SUNLIGHT was 3.3 months with an HR of 0.61 (95% CI, 0.49-0.77),^44^ which meets the ESMO, ASCO, and CCC thresholds for clinically meaningful benefit;^30^ however, in SUNLIGHT, ∼97% of participants had received only ≤2 lines of prior treatment for metastatic disease,^44^ while ∼21%-73% of patients in the RCTs included in the meta-analysis had received ≥4 prior treatments for mCRC.^22–27^ Evidence, where available, from phase II clinical trials that compared systemic monotherapies to placebo was also excluded; however, estimates from our meta-analysis are in line with outcomes seen in phase II RCTs of TAS-102 compared with placebo (absolute median difference in OS 2.4 months),^53^ and fruquintinib compared with placebo (absolute median difference in OS 2.2 months)^54^ in patients with mCRC.

As the meta-analyses focused on OS and PFS, other essential clinical aspects were not captured. In the later-line mCRC setting, patients may have additional priorities such as maintaining QoL for as long as possible and minimizing treatment-related toxicity,^30^ important considerations from the patient perspective.^28^^,^^30^^,^^55–57^ As shown in Figure S2, the therapies included in this meta-analysis have distinct toxicity profiles. These differences highlight that interpretation of survival benefit in the later-line mCRC setting should consider not only efficacy but also the expected AE profile of each treatment. Our meta-analysis did not include QoL, but analyses of CORRECT and CONCUR reported no differences in QoL between the regorafenib vs placebo arms,^23^^,^^24^ and in FRESCO-2, fruquintinib did not negatively impact QoL when compared with placebo;^58^ indicating that extending survival with active treatment can maintain QoL for longer in patients who experience clinical benefit, without negative impacts due to treatment toxicity. While current guidelines emphasize OS improvements for assessing clinically meaningful benefit, they also consider factors such as QoL when considering the magnitude of clinical benefit, as well as measures of disease activity such as PFS and disease control, highlighting the importance of considering the broad and nuanced clinical picture when assessing the magnitude of clinical benefit.^28^^,^^29^^,^^31^

## Conclusions

Our meta-analyses estimated incremental improvements in PFS and OS using multiple measures, including difference in median survival, HRs, and difference in 12-month RMST in placebo-controlled trials in the later-line mCRC setting. Considering several measures of survival in the context of later-line mCRC treatment, an incremental survival benefit with oral systemic monotherapy vs BSC alone is clinically meaningful.

## Supplementary Material

oyag175_Supplementary_Data

## Data Availability

No new data were generated or analyzed in support of this research.
