# Supplementary material for: Survival benefit of oral systemic monotherapy in previously treated metastatic colorectal cancer: a meta-analysis
Source: Oncologist. 2026 May 8;31(7):oyag175. doi: 10.1093/oncolo/oyag175 (PMC13264436; doi:10.1093/oncolo/oyag175)
Supplement: oyag175_Supplementary_Data [file oyag175_supplementary_data.docx]

# **Supplementary Material**

**Survival benefit of oral systemic monotherapy in previously treated metastatic colorectal cancer: a meta-analysis**

Per Pfeiffer, Chiara Cremolini, Michel Ducreux, Pia Osterlund, Sarah Ronnebaum, Morodoluwa Akin-Fajiye, Victoria Paly, Luis Hernandez, Elena Elez

**Table S1.** The Embase search strategy for the conduct of the SLR.

| **Search number** | **Search terms** | **Original SLR (March 2023)** | **Update (October 2023)** | **Update (June 2024)** |
| --- | --- | --- | --- | --- |
| 1 | exp colon cancer/ or exp colorectal cancer/ | 379267 | 387386 | 405737 |
| 2 | exp rectum cancer/ or exp sigmoid cancer/ | 58205 | 59706 | 62212 |
| 3 | large intestine cancer/ or cecum cancer/ | 1352 | 1374 | 1403 |
| 4 | ((colorect$ or rectal$ or rectum$ or colon or sigma$ or sigmo$ or rectosigm$ or bowel$ or anal or anus) adj3 (cancer$ or neoplas$ or oncolog$ or malignan$ or tumo?r$ or carcinoma$ or adenocarcinoma$ or metasta$ or meta-sta$ or sarcoma$ or adenom$ or lesion$)).ti,ab. | 408021 | 414786 | 432822 |
| 5 | (m-CRC or CRC or mCRC).ti,ab. | 74414 | 84685 | 91043 |
| 6 | ((cecum or cecal or caecum or caecal or il?eoc?ecal or il?eoc?ecum) adj3 (cancer$ or neoplas$ or oncolog$ or malignan$ or tumo?r$ or carcinoma$ or adenocarcinoma$ or metasta$ or meta-sta$ or sarcoma$ or adenom$ or lesion$)).ti,ab. | 3698 | 3772 | 3898 |
| 7 | (large intestin$ adj3 (cancer$ or neoplas$ or oncolog$ or malignan$ or tumo?r$ or carcinoma$ or adenocarcinoma$ or metasta$ or meta-sta$ or sarcoma$ or adenom$ or lesion$)).ti,ab. | 1841 | 1853 | 1871 |
| 8 | (lower intestin$ adj3 (cancer$ or neoplas$ or oncolog$ or malignan$ or tumo?r$ or carcinoma$ or adenocarcinoma$ or metasta$ or meta-sta$ or sarcoma$ or adenom$ or lesion$)).ti,ab. | 45 | 45 | 46 |
| 9 | or/1-8 | 507080 | 518532 | 541860 |
| 10 | tipiracil plus trifluridine/ | 1263 | 1324 | 1483 |
| 11 | ((trifluridine$ adj3 tipiracil$) or lonsurf or tas102 or tas 102 or 733030-01-8 or "tri tip" or tri?tip or FTD?TPI).ti,ab,rn,tn. | 1508 | 1625 | 1824 |
| 12 | regorafenib/ | 6474 | 6905 | 7604 |
| 13 | (regorafenib$ or resihance or stivarga or bay 73 4506 or bay 734506 or bay73 4506 or bay734506 or 1019206-88-2 or 755037-03-7 or Regonix).ti,ab,rn,tn. | 6728 | 7205 | 7919 |
| 14 | fruquintinib/ | 204 | 240 | 350 |
| 15 | (fruquintinib or elunate or “hmpl 013” or hmpl013 or 1194506-26-7).ti,ab,rn,tn. | 216 | 253 | 366 |
| 16 | (pre-treat$ or pretreat? or re-treat? or retreat? or re-challeng? or rechalleng? or re-using or reusing or re-used or reused).ti,ab. | 126072 | 128582 | 134949 |
| 17 | or/10-16 | 133639 | 136694 | 143898 |
| 18 | exp clinical trial/ or exp "clinical trial (topic)"/ or clinical trial protocol/ | 2232593 | 2259844 | 2349894 |
| 19 | multicenter study/ or "multicenter study (topic)"/ | 409112 | 414398 | 436138 |
| 20 | randomization/ | 98423 | 98547 | 99385 |
| 21 | double blind procedure/ | 208385 | 211171 | 219770 |
| 22 | single blind procedure/ | 51116 | 51932 | 55004 |
| 23 | placebo/ | 400196 | 403086 | 413630 |
| 24 | control group/ | 110913 | 110647 | 110780 |
| 25 | crossover procedure/ | 74556 | 75475 | 78255 |
| 26 | (random* or sham or placebo*).ti,ab,hw,kf. | 2515820 | 2566064 | 2680645 |
| 27 | ((singl* or doubl*) adj (blind* or dumm* or mask*)).ti,ab,hw,kf. | 358800 | 363397 | 376435 |
| 28 | ((tripl* or trebl*) adj (blind* or dumm* or mask*)).ti,ab,hw,kf. | 2085 | 2197 | 2391 |
| 29 | (control* adj3 (study or studies or trial* or group*)).ti,ab,hw,kf. | 10720028 | 10812512 | 11367689 |
| 30 | (clinical adj3 (study or studies or trial*)).ti,ab,hw,kf. | 6637894 | 6750948 | 7074564 |
| 31 | (Nonrandom* or non random* or non-random* or quasi-random* or quasirandom*).ti,ab,hw,kf. | 69316 | 70669 | 74095 |
| 32 | (phase adj3 (study or studies or trial*)).ti,ab,hw,kf. | 389155 | 396931 | 421585 |
| 33 | ((crossover or cross-over) adj3 (study or studies or trial*)).ti,ab,hw,kf. | 69119 | 69810 | 71840 |
| 34 | ((multicent* or multi-cent*) adj3 (study or studies or trial*)).ti,ab,hw,kf. | 515085 | 523576 | 551778 |
| 35 | allocated.ti,ab,hw. | 107786 | 110121 | 115371 |
| 36 | ((open label or open-label) adj5 (study or studies or trial*)).ti,ab,hw,kf. | 85709 | 87031 | 92501 |
| 37 | ((equivalence or superiority or non-inferiority or noninferiority) adj3 (study or studies or trial*)).ti,ab,hw,kf. | 18026 | 18518 | 20001 |
| 38 | (pragmatic study or pragmatic studies).ti,ab,hw,kf. | 885 | 936 | 1017 |
| 39 | ((pragmatic or practical) adj3 trial*).ti,ab,hw,kf. | 8520 | 8815 | 9579 |
| 40 | ((quasiexperimental or quasi-experimental) adj3 (study or studies or trial*)).ti,ab,hw,kf. | 18994 | 20003 | 21602 |
| 41 | trial.ti,kf. | 435271 | 441608 | 466695 |
| 42 | or/18-41 | 14581057 | 14749186 | 15459078 |
| 43 | observational study/ | 320042 | 338443 | 376129 |
| 44 | cohort analysis/ | 1008052 | 1054540 | 1172581 |
| 45 | longitudinal study/ | 190232 | 197952 | 214422 |
| 46 | follow up/ | 2017607 | 2078759 | 2198369 |
| 47 | retrospective study/ | 1435962 | 1495817 | 1629743 |
| 48 | exp case control study/ | 221978 | 225993 | 236851 |
| 49 | cross-sectional study/ | 550801 | 583870 | 639759 |
| 50 | quasi experimental study/ | 11041 | 11602 | 12579 |
| 51 | prospective study/ | 860936 | 882819 | 921274 |
| 52 | (observational adj3 (study or studies or design or analysis or analyses)).ti,ab,kf. | 341650 | 353185 | 381087 |
| 53 | cohort*.ti,ab,kf. | 1446043 | 1484129 | 1593213 |
| 54 | (prospective adj7 (study or studies or design or analysis or analyses)).ti,ab,kf. | 804603 | 820063 | 861812 |
| 55 | ((follow up or followup) adj7 (study or studies or design or analysis or analyses)).ti,ab,kf. | 267490 | 272520 | 285700 |
| 56 | ((longitudinal or longterm or (long adj term)) adj7 (study or studies or design or analysis or analyses or data)).ti,ab,kf. | 488395 | 499364 | 525997 |
| 57 | (retrospective adj7 (study or studies or design or analysis or analyses or data or review)).ti,ab,kf. | 1130234 | 1162396 | 1245385 |
| 58 | ((case adj control) or (case adj comparison) or (case adj controlled)).ti,ab,kf. | 212110 | 214802 | 223694 |
| 59 | (case-referent adj3 (study or studies or design or analysis or analyses)).ti,ab,kf. | 703 | 705 | 712 |
| 60 | (population adj3 (study or studies or analysis or analyses)).ti,ab,kf. | 349025 | 356161 | 377363 |
| 61 | (descriptive adj3 (study or studies or design or analysis or analyses)).ti,ab,kf. | 162738 | 170780 | 182820 |
| 62 | ((multidimensional or (multi adj dimensional)) adj3 (study or studies or design or analysis or analyses)).ti,ab,kf. | 5730 | 5885 | 6233 |
| 63 | (cross adj sectional adj7 (study or studies or design or research or analysis or analyses or survey or findings)).ti,ab,kf. | 564016 | 587402 | 631773 |
| 64 | ((natural adj experiment) or (natural adj experiments)).ti,ab,kf. | 3528 | 3671 | 3938 |
| 65 | (quasi adj (experiment or experiments or experimental)).ti,ab,kf. | 24881 | 25870 | 27740 |
| 66 | ((non experiment or nonexperiment or non experimental or nonexperimental) adj3 (study or studies or design or analysis or analyses)).ti,ab,kf. | 2330 | 2405 | 2501 |
| 67 | (prevalence adj3 (study or studies or analysis or analyses)).ti,ab,kf. | 71101 | 72948 | 77035 |
| 68 | or/43-67 | 6312767 | 6495905 | 6895225 |
| 69 | 9 and 17 and (42 or 68) | 5312 | 5547 | 6027 |
| 70 | animal/ or animal experiment/ or (rat or rats or mouse or mice or murine or rodent or rodents or hamster or hamsters or pig or pigs or porcine or rabbit or rabbits or animal or animals or dogs or dog or cats or cow or bovine or sheep or ovine or monkey or monkeys).ti,ab,ot,hw. | 7568080 | 7654932 | 7856384 |
| 71 | exp human/ or human experiment/ | 25231417 | 25614733 | 26632583 |
| 72 | 70 not (70 and 71) | 5686821 | 5741193 | 5856783 |
| 73 | (case report or case study or case series).ti. or case reports/ or case study/ or case report$.jx. or case report$.jw. or (news or “newspaper article” or note or editorial or comment or letter or overall or short survey or tombstone or books).pt. or in vitro Techniques/ or in vitro study/ or (commentary or editorial or comment or letter).ti. | 7251709 | 5665906 | 5855164 |
| 74 | review.pt. not (systematic or (meta and analy$) or ((indirect or mixed) and treatment comparison)).ti,ab. | 2828911 | 2880139 | 2965991 |
| 75 | 69 not (72 or 73 or 74) | 4094 | 4257 | 4627 |
| 76 | ("conference abstract" or "conference review" or "conference paper").pt. or conference$.so,st. | 5515501 | 5732717 | 5995918 |
| 77 | 75 not 76 | 1991 | 2053 | 2250 |
| 78 | limit 76 to yr=”2020-current” | 886226 | 1079130 | 1321375 |
| 79 | 75 and 78 | 609 | 688 | 858 |
| 80 | **77 or 79** | **2600** | **2741** | 3108 |
| 81 | ("202335" or "202336" or "202337" or "202338" or "202339" or 20234$ or 20235$ or 2024$).em. or (202309$ or 202310$ or 202311$ or 202312$ or 2024$).dc. |  |  | 2120690 |
| 82 | 80 and 81 |  |  | 480 |

Note: the inclusion criteria for the SLR were broader than those of the meta-analyses.

Abbreviations (excluding search terms): SLR, systematic literature review.

**Table S2**. The MEDLINE search strategy for the conduct of the SLR.

| **Search number** | **Search terms** | **Original SLR (March 2023)** | **Update (October 2023)** | **Update (June 2024)** |
| --- | --- | --- | --- | --- |
| 1 | exp colorectal neoplasms/ | 234140 | 239485 | 246436 |
| 2 | exp Cecal Neoplasms/ | 6094 | 6162 | 6275 |
| 3 | ((colorect$ or rectal$ or rectum$ or colon or sigma$ or sigmo$ or rectosigm$ or bowel$ or anal or anus) adj3 (cancer$ or neoplas$ or oncolog$ or malignan$ or tumo?r$ or carcinoma$ or adenocarcinoma$ or metasta$ or meta-sta$ or sarcoma$ or adenom$ or lesion$)).ti,ab. | 274470 | 283790 | 295046 |
| 4 | (m-CRC or CRC or mCRC).ti,ab. | 44979 | 51233 | 55139 |
| 5 | ((cecum or cecal or caecum or caecal or il?eoc?ecal or il?eoc?ecum) adj3 (cancer$ or neoplas$ or oncolog$ or malignan$ or tumo?r$ or carcinoma$ or adenocarcinoma$ or metasta$ or meta-sta$ or sarcoma$ or adenom$ or lesion$)).ti,ab. | 2827 | 2874 | 2961 |
| 6 | (large intestin$ adj3 (cancer$ or neoplas$ or oncolog$ or malignan$ or tumo?r$ or carcinoma$ or adenocarcinoma$ or metasta$ or meta-sta$ or sarcoma$ or adenom$ or lesion$)).ti,ab. | 1858 | 1866 | 1872 |
| 7 | (lower intestin$ adj3 (cancer$ or neoplas$ or oncolog$ or malignan$ or tumo?r$ or carcinoma$ or adenocarcinoma$ or metasta$ or meta-sta$ or sarcoma$ or adenom$ or lesion$)).ti,ab. | 35 | 35 | 35 |
| 8 | or/1-7 | 335394 | 345592 | 357816 |
| 9 | Trifluridine/ | 698 | 733 | 773 |
| 10 | ((trifluridine$ adj3 tipiracil$) or lonsurf or tas102 or tas 102 or 733030-01-8 or "tri tip" or tri?tip or FTD?TPI).ti,ab,rn. | 483 | 542 | 596 |
| 11 | (regorafenib$ or resihance or stivarga or bay 73 4506 or bay 734506 or bay73 4506 or bay734506 or 1019206-88-2 or 755037-03-7 or Regonix).ti,ab,rn. | 1773 | 1892 | 2031 |
| 12 | (fruquintinib or elunate or "hmpl 013" or hmpl013 or 1194506-26-7).ti,ab,rn. | 77 | 91 | 122 |
| 13 | (pre-treat$ or pretreat? or re-treat? or retreat? or re-challeng? or rechalleng? or re-using or reusing or re-used or reused).ti,ab. | 76213 | 79173 | 82686 |
| 14 | or/9-13 | 78713 | 81845 | 85547 |
| 15 | (Randomized Controlled Trial or Controlled Clinical Trial or Pragmatic Clinical Trial or Clinical Study or Adaptive Clinical Trial or Equivalence Trial).pt. | 686347 | 698763 | 712726 |
| 16 | (Clinical Trial or Clinical Trial, Phase I or Clinical Trial, Phase II or Clinical Trial, Phase III or Clinical Trial, Phase IV or Clinical Trial Protocol).pt. | 608673 | 613218 | 618645 |
| 17 | Multicenter Study.pt. | 331785 | 338490 | 347675 |
| 18 | Clinical Studies as Topic/ | 784 | 801 | 836 |
| 19 | exp Clinical Trial/ or exp Clinical Trials as Topic/ or Clinical Trial Protocol/ or Clinical Trial Protocols as Topic/ | 1267395 | 1285813 | 1309689 |
| 20 | Multicenter Study/ or Multicenter Studies as Topic/ | 350913 | 358271 | 368937 |
| 21 | Random Allocation/ | 106910 | 106969 | 107284 |
| 22 | Double-Blind Method/ | 174649 | 176258 | 178845 |
| 23 | Single-Blind Method/ | 32577 | 32959 | 33551 |
| 24 | Placebos/ | 35925 | 35932 | 35966 |
| 25 | Control Groups/ | 1923 | 2047 | 2130 |
| 26 | Cross-Over Studies/ | 54838 | 55623 | 56799 |
| 27 | (random* or sham or placebo*).ti,ab,hw,kf. | 1785280 | 1841249 | 1913480 |
| 28 | ((singl* or doubl*) adj (blind* or dumm* or mask*)).ti,ab,hw,kf. | 265108 | 269787 | 276093 |
| 29 | ((tripl* or trebl*) adj (blind* or dumm* or mask*)).ti,ab,hw,kf. | 1550 | 1675 | 1843 |
| 30 | (control* adj3 (study or studies or trial* or group*)).ti,ab,hw,kf. | 1899857 | 1949125 | 2012251 |
| 31 | (clinical adj3 (study or studies or trial*)).ti,ab,hw,kf. | 1414937 | 1449059 | 1492000 |
| 32 | (Nonrandom* or non random* or non-random* or quasi-random* or quasirandom*).ti,ab,hw,kf. | 53609 | 55646 | 58177 |
| 33 | (phase adj3 (study or studies or trial*)).ti,ab,hw,kf. | 175409 | 180709 | 187162 |
| 34 | ((crossover or cross-over) adj3 (study or studies or trial*)).ti,ab,hw,kf. | 76056 | 77470 | 79282 |
| 35 | ((multicent* or multi-cent*) adj3 (study or studies or trial*)).ti,ab,hw,kf. | 402991 | 413322 | 427596 |
| 36 | allocated.ti,ab,hw. | 82251 | 85324 | 89372 |
| 37 | ((open label or open-label) adj5 (study or studies or trial*)).ti,ab,hw,kf. | 43816 | 45713 | 48053 |
| 38 | ((equivalence or superiority or non-inferiority or noninferiority) adj3 (study or studies or trial*)).ti,ab,hw,kf. | 11751 | 12483 | 13433 |
| 39 | (pragmatic study or pragmatic studies).ti,ab,hw,kf. | 579 | 616 | 652 |
| 40 | ((pragmatic or practical) adj3 trial*).ti,ab,hw,kf. | 7511 | 7892 | 8483 |
| 41 | ((quasiexperimental or quasi-experimental) adj3 (study or studies or trial*)).ti,ab,hw,kf. | 11800 | 12662 | 13823 |
| 42 | trial.ti,kf. | 301746 | 315854 | 334013 |
| 43 | or/15-42 | 3744136 | 3851873 | 3991209 |
| 44 | Epidemiologic Methods/ | 31613 | 31619 | 31621 |
| 45 | exp Epidemiologic Studies/ | 3096123 | 3180276 | 3294029 |
| 46 | Observational Studies as Topic/ | 8593 | 9066 | 9815 |
| 47 | Clinical Studies as Topic/ | 784 | 801 | 836 |
| 48 | single-case studies as topic/ | 98 | 99 | 109 |
| 49 | (Observational Study or Validation Studies or Clinical Study).pt. | 144965 | 152519 | 162786 |
| 50 | (observational adj3 (study or studies or design or analysis or analyses)).ti,ab,kf. | 213488 | 227573 | 245514 |
| 51 | cohort*.ti,ab,kf. | 834083 | 884499 | 948854 |
| 52 | (prospective adj7 (study or studies or design or analysis or analyses)).ti,ab,kf. | 523560 | 543003 | 567446 |
| 53 | ((follow up or followup) adj7 (study or studies or design or analysis or analyses)).ti,ab,kf. | 166861 | 172661 | 180035 |
| 54 | ((longitudinal or longterm or (long adj term)) adj7 (study or studies or design or analysis or analyses or data)).ti,ab,kf. | 339090 | 353566 | 372525 |
| 55 | (retrospective adj7 (study or studies or design or analysis or analyses or data or review)).ti,ab,kf. | 662636 | 700245 | 749956 |
| 56 | ((case adj control) or (case adj comparison) or (case adj controlled)).ti,ab,kf. | 156908 | 162142 | 168569 |
| 57 | (case-referent adj3 (study or studies or design or analysis or analyses)).ti,ab,kf. | 636 | 639 | 642 |
| 58 | (population adj3 (study or studies or analysis or analyses)).ti,ab,kf. | 228783 | 238048 | 249761 |
| 59 | (descriptive adj3 (study or studies or design or analysis or analyses)).ti,ab,kf. | 106266 | 112539 | 120817 |
| 60 | ((multidimensional or (multi adj dimensional)) adj3 (study or studies or design or analysis or analyses)).ti,ab,kf. | 4843 | 5060 | 5363 |
| 61 | (cross adj sectional adj7 (study or studies or design or research or analysis or analyses or survey or findings)).ti,ab,kf. | 422944 | 449988 | 485462 |
| 62 | ((natural adj experiment) or (natural adj experiments)).ti,ab,kf. | 3184 | 3421 | 3701 |
| 63 | (quasi adj (experiment or experiments or experimental)).ti,ab,kf. | 19759 | 21034 | 22711 |
| 64 | ((non experiment or nonexperiment or non experimental or nonexperimental) adj3 (study or studies or design or analysis or analyses)).ti,ab,kf. | 1677 | 1749 | 1818 |
| 65 | (prevalence adj3 (study or studies or analysis or analyses)).ti,ab,kf. | 48582 | 50643 | 53401 |
| 66 | or/44-65 | 4265263 | 4419247 | 4617854 |
| 67 | 8 and 14 and (43 or 66) | 1227 | 1307 | 1413 |
| 68 | exp animals/ not humans/ | 5104362 | 5160002 | 5228227 |
| 69 | case reports/ or case study/ or case report$.jw. or (case report or case series).ti. | 2888967 | 2452143 | 2504052 |
| 70 | (“Introductory Journal Article” or letter or News or “Newspaper Article” or Editorial or Comment or Overall).pt. or in vitro Techniques/ or (commentary or editorial or comment or letter).ti. | 3999628 | 2930932 | 2995930 |
| 71 | review.pt. not (systematic or (meta and analy$) or ((indirect or mixed) and treatment comparison)).ti,ab. | 2907475 | 2984452 | 3082296 |
| 72 | **67 not (68 or 69 or 70 or 71)** | **1020** | **1101** | 1194 |
| 73 | (202309$ or 202310$ or 202311$ or 202312$ or 2024$).ed,dt. | - | - | 1360424 |
| 74 | 72 and 73 | - | - | 129 |

Note: the inclusion criteria for the SLR were broader than those of the meta-analyses.

Abbreviations (excluding search terms): SLR, systematic literature review.

**Table S3.** The CDSR search strategy for the conduct of the SLR.

| **Search Number** | **Search Terms** | **Original SLR (March 2023)** | **Update (October 2023)** | **Update (June 2024)** |
| --- | --- | --- | --- | --- |
| 1 | ((colorect$ or rectal$ or rectum$ or colon or sigma$ or sigmo$ or rectosigm$ or bowel$ or anal or anus) adj3 (cancer$ or neoplas$ or oncolog$ or malignan$ or tumo?r$ or carcinoma$ or adenocarcinoma$ or metasta$ or meta-sta$ or sarcoma$ or adenom$ or lesion$)).ti,ab,kw. | 152 | 153 | 155 |
| 2 | (m-CRC or CRC or mCRC).ti,ab,kw. | 13 | 14 | 14 |
| 3 | ((cecum or cecal or caecum or caecal or il?eoc?ecal or il?eoc?ecum) adj3 (cancer$ or neoplas$ or oncolog$ or malignan$ or tumo?r$ or carcinoma$ or adenocarcinoma$ or metasta$ or meta-sta$ or sarcoma$ or adenom$ or lesion$)).ti,ab,kw. | 0 | 0 | 0 |
| 4 | (large intestin$ adj3 (cancer$ or neoplas$ or oncolog$ or malignan$ or tumo?r$ or carcinoma$ or adenocarcinoma$ or metasta$ or meta-sta$ or sarcoma$ or adenom$ or lesion$)).ti,ab,kw. | 0 | 0 | 0 |
| 5 | (lower intestin$ adj3 (cancer$ or neoplas$ or oncolog$ or malignan$ or tumo?r$ or carcinoma$ or adenocarcinoma$ or metasta$ or meta-sta$ or sarcoma$ or adenom$ or lesion$)).ti,ab,kw. | 0 | 0 | 0 |
| 6 | or/1-5 | 152 | 153 | 155 |
| 7 | ((trifluridine$ adj3 tipiracil$) or lonsurf or tas102 or tas 102 or 733030-01-8 or "tri tip" or tri?tip or FTD?TPI).af. | 4 | 4 | 4 |
| 8 | (regorafenib$ or resihance or stivarga or bay 73 4506 or bay 734506 or bay73 4506 or bay734506 or 1019206-88-2 or 755037-03-7 or Regonix).af. | 9 | 10 | 10 |
| 9 | (fruquintinib or elunate or "hmpl 013" or hmpl013 or 1194506-26-7).af. | 0 | 0 | 0 |
| 10 | (pre-treat$ or pretreat? or re-treat? or retreat? or re-challeng? or rechalleng? or re-using or reusing or re-used or reused).af. | 567 | 570 | 574 |
| 11 | or/7-10 | 573 | 576 | 580 |
| 12 | **6 and 11** | **10** | **9*** | 9 |
| 13 | (202309$ or 202310$ or 202311$ or 202312$ or 2024$).up. | - | - | 2641 |
| 14 | 12 and 13 | - | - | 1 |

Note: the inclusion criteria for the SLR were broader than those of the meta-analyses.

*Difference in total between March and October 2023 searches reflects removal of an SLR by CDSR which was not updated by CDSR by the time of the October 2023 searches. Neither the original nor updated SLR is relevant (i.e., would meet the inclusion criteria) for this study.

Abbreviations (excluding search terms): CDSR, Cochrane Database of Systematic Reviews; SLR, systematic literature review.

**Table S4.** The Cochrane Library Central Register of Controlled Trials.

| **Search Number** | **Search Terms** | **Original SLR (March 2023)** | **Update (October 2023)** | **Update (June 2024)** |
| --- | --- | --- | --- | --- |
| 1 | exp colorectal neoplasms/ | 10865 | 11142 | 12740 |
| 2 | exp Cecal Neoplasms/ | 27 | 28 | 38 |
| 3 | ((colorect$ or rectal$ or rectum$ or colon or sigma$ or sigmo$ or rectosigm$ or bowel$ or anal or anus) adj3 (cancer$ or neoplas$ or oncolog$ or malignan$ or tumo?r$ or carcinoma$ or adenocarcinoma$ or metasta$ or meta-sta$ or sarcoma$ or adenom$ or lesion$)).ti,ab. | 23704 | 24586 | 25920 |
| 4 | (m-CRC or CRC or mCRC).ti,ab. | 3458 | 5447 | 5862 |
| 5 | ((cecum or cecal or caecum or caecal or il?eoc?ecal or il?eoc?ecum) adj3 (cancer$ or neoplas$ or oncolog$ or malignan$ or tumo?r$ or carcinoma$ or adenocarcinoma$ or metasta$ or meta-sta$ or sarcoma$ or adenom$ or lesion$)).ti,ab. | 95 | 103 | 121 |
| 6 | (large intestin$ adj3 (cancer$ or neoplas$ or oncolog$ or malignan$ or tumo?r$ or carcinoma$ or adenocarcinoma$ or metasta$ or meta-sta$ or sarcoma$ or adenom$ or lesion$)).ti,ab. | 61 | 61 | 62 |
| 7 | (lower intestin$ adj3 (cancer$ or neoplas$ or oncolog$ or malignan$ or tumo?r$ or carcinoma$ or adenocarcinoma$ or metasta$ or meta-sta$ or sarcoma$ or adenom$ or lesion$)).ti,ab. | 1 | 1 | 1 |
| 8 | or/1-7 | 25845 | 26835 | 28409 |
| 9 | Trifluridine/ | 109 | 118 | 150 |
| 10 | ((trifluridine$ adj3 tipiracil$) or lonsurf or tas102 or tas 102 or 733030-01-8 or "tri tip" or tri?tip or FTD?TPI).ti,ab. | 301 | 329 | 370 |
| 11 | (regorafenib$ or resihance or stivarga or bay 73 4506 or bay 734506 or bay73 4506 or bay734506 or 1019206-88-2 or 755037-03-7 or Regonix).ti,ab. | 617 | 642 | 692 |
| 12 | (fruquintinib or elunate or "hmpl 013" or hmpl013 or 1194506-26-7).ti,ab. | 54 | 57 | 75 |
| 13 | (pre-treat$ or pretreat? or re-treat? or retreat? or re-challeng? or rechalleng? or re-using or reusing or re-used or reused).ti,ab. | 10728 | 11119 | 11874 |
| 14 | or/9-13 | 11610 | 12042 | 12874 |
| 15 | 8 and 14 | 746 | 797 | 874 |
| 16 | limit 15 to trial registry record | 234 | 245 | 260 |
| 17 | 15 not 16 | 512 | 552 | 614 |
| 18 | limit 17 to (conference proceeding and yr="1898 - 2019") | 261 | 238 | 242 |
| 19 | 17 not 18 | 251 | 314 | 372 |
| 20 | limit 19 to yr="2023 -Current" | - | - | 95 |

Note: the inclusion criteria for the SLR were broader than those of the meta-analyses.

Abbreviations (excluding search terms): SLR, systematic literature review.

**Table S5.** Quality assessment of RCTs by Cochrane Risk-of-Bias assessment tool 2.0.

| **Study name** | Bias arising from the randomization process | Bias due to deviations from intended interventions | Bias due to missing outcome data | Bias in measurement of the outcome | Bias in selection of the reported result | Overall bias |
| --- | --- | --- | --- | --- | --- | --- |
| **CONCUR**^1^ | Low  concerns | Low concerns | Low concerns | Low  concerns | Low concerns | Low concerns |
| **CORRECT**^2^ | Low  concerns | Low concerns | Low concerns | Low  concerns | Low concerns | Low concerns |
| **FRESCO**^3^ | Low  concerns | Low concerns | Low concerns | Low  concerns | Low concerns | Low concerns |
| **FRESCO-2**^4^ | Low  concerns | Low concerns | Low concerns | Low  concerns | Low concerns | Low concerns |
| **RECOURSE**^5,6^ | Low  concerns | Low concerns | Low concerns | Low  concerns | Low concerns | Some concerns |
| **TERRA**^7^ | Low  concerns | Low concerns | Low concerns | Low  concerns | Low concerns | Low concerns |

Abbreviations: RCT, randomized controlled trial.

**Table S6.** Data on prior treatments in the studies included in the meta-analyses.

| **Trial,  start of enrollment** | **Trial arm** | ***n*** | **Prior lines of treatment, %** | **Prior targeted therapies** | | **Prior oral systemic monotherapies** | | |
| --- | --- | --- | --- | --- | --- | --- | --- | --- |
|  |  |  |  | **Previous  anti-VEGF, %** | **Previous  anti-EGFR, %** | **Prior regorafenib only, %** | **Prior TAS-102 only, %** | **Both prior regorafenib and TAS-102, %** |
| **CONCUR, 2012**^1^ | Regorafenib | 136 | For metastatic disease:  1–2: 35*^†^  3: 24* ≥4: 38* | Anti-VEGF but not anti-EGFR: 24  Anti-VEGF and anti-EGFR: 18 | Anti-EGFR but not anti-VEGF:  18  Anti-EGFR and anti-VEGF: 18 | – | – | – |
|  | Placebo | 68 | For metastatic disease:  1–2: 35* 3: 25* ≥4: 40* | Anti-VEGF but not anti-EGFR: 19  Anti-VEGF and anti-EGFR: 18 | Anti-EGFR but not anti-VEGF: 25  Anti-EGFR and anti-VEGF: 18 | – | – | – |
| **CORRECT, 2010**^2^ | Regorafenib | 505 | For metastatic disease:  1–2: 27*^†^ 3: 25* ≥4: 49* | 100 | Patients stopping prior panitumumab or cetuximab or both due to progression: 43 | – | – | – |
|  | Placebo | 255 | For metastatic disease:  1–2: 25*^†^ 3: 28* ≥4: 47* | 100 | Patients stopping prior panitumumab or cetuximab or both due to progression: 42 | – | – | – |
| **FRESCO, 2014**^3^ | Fruquintinib | 278 | For metastatic disease:  ≤3: 79* ≥4: 21* | 30 | 14 | – | – | – |
|  | Placebo | 138 | For metastatic disease:  ≤3: 78* ≥4: 22* | 30 | 14 | – | – | – |
| **FRESCO-2, 2020**^4^ | Fruquintinib | 461 | For metastatic disease:  ≤3: 27* ≥4: 73* | 97 | 39 | 9 | 52 | 39 |
|  | Placebo | 230 | For metastatic disease:  ≤3: 28* ≥4: 72* | 96 | 38 | 8 | 53 | 40 |
| **RECOURSE, 2012**^5^ | TAS-102 | 534 | Treatment intent not specified: 2: 18 3: 22 ≥4: 60 | Bevacizumab: 100 | 52 | 17 | – | – |
|  | Placebo | 266 | Treatment intent not specified:  2: 17 3: 20 ≥4: 63 | Bevacizumab: >99% | 54 | 20 | – | – |
| **TERRA, 2013**^7^ | TAS-102 | 271 | Treatment intent not specified:  2: 23 3: 27 ≥4: 50 | Anti-VEGF but not anti-EGFR: 19  Anti-VEGF and anti-EGFR: 9 | Anti-EGFR but not anti-VEGF: 17  Anti-EGFR and anti-VEGF: 9 | – | – | – |
|  | Placebo | 135 | Treatment intent not specified:  2: 19 3: 27 ≥4: 55 | Anti-VEGF but not anti-EGFR: 20  Anti-VEGF and anti-EGFR: 13 | Anti-EGFR but not anti-VEGF: 19  Anti-EGFR but not anti-VEGF: 13 | – | – | – |

Dashes (–) indicate not reported or not applicable (some therapies were not available at the time that some studies were initiated).

*Prior lines of therapy in metastatic disease. ^†^In CONCUR, four patients (3%) in the regorafenib group had not previously received any treatment for metastatic disease;^1^ in CORRECT, five patients (2%) in the placebo group and 16 patients (3%) in the regorafenib group had received only one previous line of therapy for metastatic disease.^2^

Abbreviations: EGFR, epidermal growth factor receptor; TAS-102, trifluridine/tipiracil; VEGF, vascular endothelial growth factor receptor.

**Figure S1.** Sensitivity analyses excluding FRESCO-2 for meta-analyses of A. HRs for OS, B. difference in 12-month RMSTs for OS, C. HRs for PFS, D. difference in 12‑month RMSTs for PFS.

**
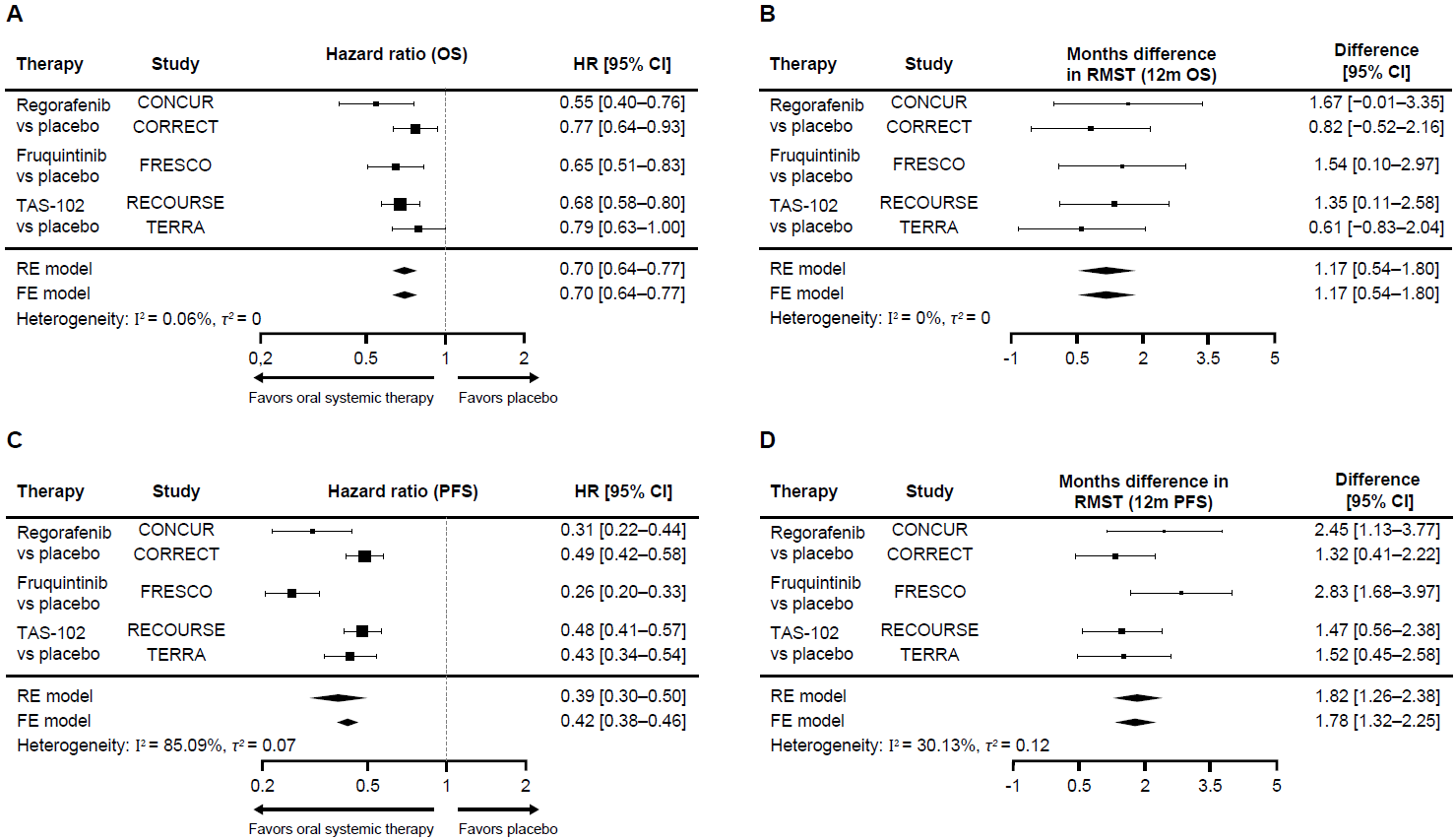
**

Abbreviations: CI, confidence interval; FE, fixed effect; HR, hazard ratio; OS, overall survival; PFS, progression-free survival; RE, random effects; RMST, restricted mean survival time; TAS-102, trifluridine/tipiracil.

**Figure S2.** Percentage of patients experiencing grade 3–4 adverse events in FRESCO,^3^ FRESCO-2,^4^ RECOURSE,^5^ TERRA,^7^ CORRECT,^2^ and CONCUR^1^ (≥5% in any arm).


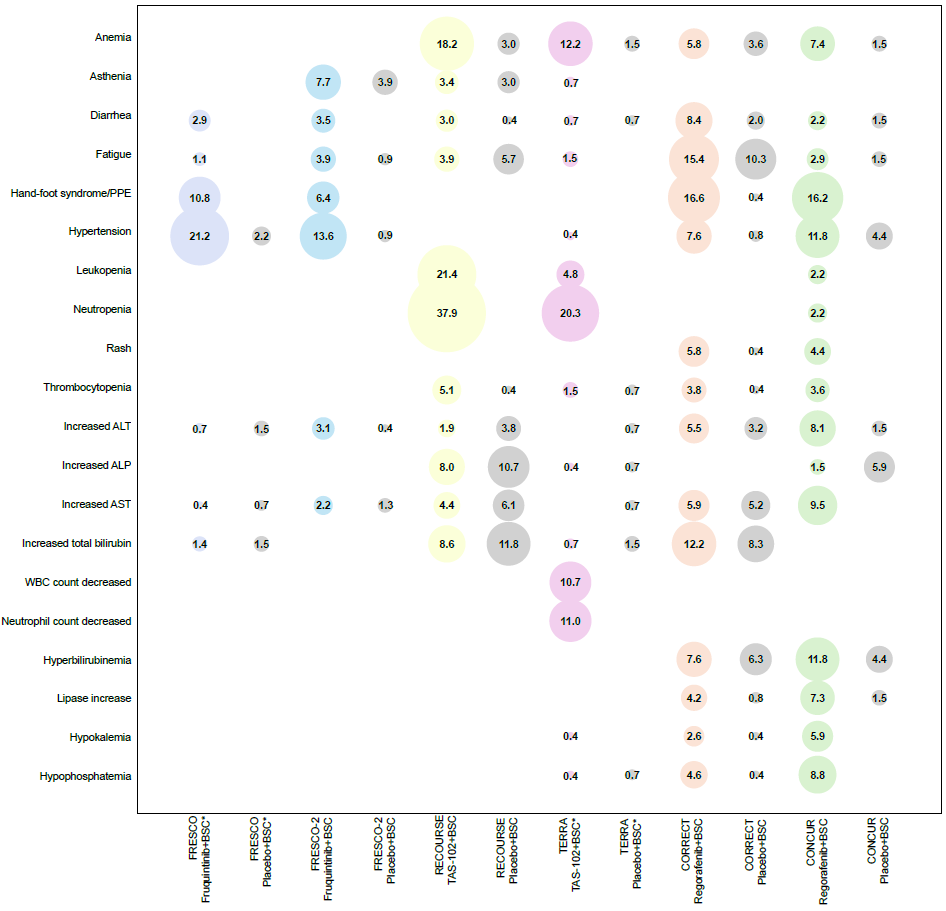


*Treatment-related.

Abbreviations: ALP, alkaline phosphatase; ALT, alanine aminotransferase; AST, aspartate aminotransferase; BSC, best supportive care; PPE, palmar-plantar erythrodysesthesia; WBC, white blood cell.

**Supplementary References**

1. Li J, Qin S, Xu R, et al. Regorafenib plus best supportive care versus placebo plus best supportive care in Asian patients with previously treated metastatic colorectal cancer (CONCUR): a randomised, double-blind, placebo-controlled, phase 3 trial. *Lancet Oncol.* 2015;16:619-629
2. Grothey A, Van Cutsem E, Sobrero A, et al. Regorafenib monotherapy for previously treated metastatic colorectal cancer (CORRECT): an international, multicentre, randomised, placebo-controlled, phase 3 trial. *Lancet.* 2013;381:303-312
3. Li J, Qin S, Xu RH, et al. Effect of fruquintinib vs placebo on overall survival in patients with previously treated metastatic colorectal cancer: the FRESCO randomized clinical trial. *JAMA*. 2018;319:2486-2496
4. Dasari A, Lonardi S, Garcia-Carbonero R, et al. Fruquintinib versus placebo in patients with refractory metastatic colorectal cancer (FRESCO-2): an international, multicentre, randomised, double-blind, phase 3 study. *Lancet*. 2023;402:41-53
5. Mayer RJ, Van Cutsem E, Falcone A, et al. Randomized trial of TAS-102 for refractory metastatic colorectal cancer. *N Eng J Med*. 2015;372:1909-1919
6. Tabernero J, Argiles G, Sobrero AF, et al. Effect of trifluridine/tipiracil in patients treated in RECOURSE by prognostic factors at baseline: an exploratory analysis. *ESMO Open*. 2020;5:e000752
7. Xu J, Kim TW, Shen L, et al. Results of a randomized, double-blind, placebo-controlled, phase III trial of trifluridine/tipiracil (TAS-102) monotherapy in Asian patients with previously treated metastatic colorectal cancer: the TERRA study. *J Clin Oncol.* 2018;36:350-358
